# Small Organic Compounds Mimicking the Effector Domain of Myristoylated Alanine-Rich C-Kinase Substrate Stimulate Female-Specific Neurite Outgrowth

**DOI:** 10.3390/ijms241814271

**Published:** 2023-09-19

**Authors:** Monica Tschang, Suneel Kumar, Wise Young, Melitta Schachner, Thomas Theis

**Affiliations:** 1 Keck Center for Collaborative Neuroscience and Department of Cell Biology and Neuroscience, Rutgers University, Piscataway, NJ 08554, USA; mat361@scarletmail.rutgers.edu (M.T.); wisey@dls.rutgers.edu (W.Y.); 2 Department of Biomedical Engineering, Rutgers, The State University of New Jersey, Piscataway, NJ 08844, USA; sk1350@soe.rutgers.edu

**Keywords:** MARCKS, ED peptide, mimetics, small compound libraries, sex specificity, neurite outgrowth, polysialic acid

## Abstract

Myristoylated alanine-rich C-kinase substrate (MARCKS) is a critical member of a signaling cascade that influences disease-relevant neural functions such as neural growth and plasticity. The effector domain (ED) of MARCKS interacts with the extracellular glycan polysialic acid (PSA) through the cell membrane to stimulate neurite outgrowth in cell culture. We have shown that a synthetic ED peptide improves functional recovery after spinal cord injury in female but not male mice. However, peptides themselves are unstable in therapeutic applications, so we investigated more pharmacologically relevant small organic compounds that mimic the ED peptide to maximize therapeutic potential. Using competition ELISAs, we screened small organic compound libraries to identify molecules that structurally and functionally mimic the ED peptide of MARCKS. Since we had shown sex-specific effects of MARCKS on spinal cord injury recovery, we assayed neuronal viability as well as neurite outgrowth from cultured cerebellar granule cells of female and male mice separately. We found that epigallocatechin, amiodarone, sertraline, tegaserod, and nonyloxytryptamine bind to a monoclonal antibody against the ED peptide, and compounds stimulate neurite outgrowth in cultured cerebellar granule cells of female mice only. Therefore, a search for compounds that act in males appears warranted.

## 1. Introduction

We had previously identified small organic compounds that are part of the human diet or are FDA-approved molecules that mimic regenerative-beneficial properties after neurotrauma [1,2,3]. We followed up on these results to investigate these compounds in more detail regarding the functions of adhesion molecules, glycans, and their binding partners. Cell adhesion molecules and their associated glycans are important players in the development of the nervous system [4,5]. One of these glycans is polysialic acid (PSA), a long homopolymer of α2,8-linked N-acetylneuraminic acid units, which is essential in neural development [6]. PSA is a post-translational modification generated by two polysialyltransferases [7]. PSA is predominantly attached to the neural cell adhesion molecule (NCAM) but also can be a posttranslational modification on other glycoproteins such as SynCAM-1, polysialyltransferase ST8SiaII, neuropilin-2, chemokine receptor CCR7, and scavenger receptor CD36 [6,8,9]. PSA promotes important neuronal functions such as neurite outgrowth and cell migration [10]. Peptides and small organic compounds that were identified as structural and functional mimetics of PSA improved functional recovery after neurotrauma [11,12,13,14]. PSA is also overexpressed in various tumors, and its expression enhances tumor cell migration and invasion [15,16,17,18]. In addition, PSA is expressed in the immune system and plays an important role in its innate and adaptive responses [19]. Given its important role in human health, PSA is an attractive target for drug discovery [20,21]. In this context, it is interesting that brain-derived neurotrophic factor [22] and fibroblast growth factor 2 [23] bind to PSA. In addition, via an anti-idiotypic antibody of PSA utilized in affinity chromatography, we identified several receptors for PSA, such as histone H1 [24], cofilin [25], PC4 [25], and myristoylated alanine-rich C-kinase substrate (MARCKS) [26].

The focus of the present study is on MARCKS, an intracellular membrane-bound protein [26] and one of the major substrates for protein kinase C with its phosphorylation sites in the functionally essential effector domain (ED) of 25 amino acids [27,28]. The N-terminal myristoyl group dips MARCKS into the plasma membrane from the cytoplasmic side [29], where it can be reached by extracellular PSA, as we have published. We suggest that the positively charged lysine residues of the ED electrostatically interact with negatively charged extracellular PSA in the plasma membrane. It has often been suggested that even negatively charged carbohydrates can exhibit hydrophobic properties in addition to the hydrophilic ones. This feature could contribute to the interactions of PSA with the ED peptide [30]. We have shown that extracellular PSA interacts with the ED of intracellular MARCKS through or at the plasma membrane [26]. A synthetic peptide derived from the ED of MARCKS interferes with the interaction between MARCKS and membrane-bound PSA-carrying NCAM at the cell surface and enhances neurite outgrowth in vitro [26]. Remarkably, ED peptide and the here-identified small organic compounds enhance the neurite outgrowth of female neurons only. Similarly, we identified that ED peptide improves functional recovery after spinal cord injury in female mice only [31].

In the present study, we identified epigallocatechin, amiodarone, sertraline, nonyloxytryptamine, and tegaserod as structural and functional mimetics of the ED peptide. These compounds bind to PSA and enhance neurite outgrowth of cerebellar granule cells from female but not male mice. Based on the Human Protein Atlas, MARCKS and PSA are broadly expressed in many human tissues [32]. Thus, we thought it reasonable to focus on the question of whether ED peptide mimetics affect only female, but not male, ED-related neuronal functions.

## 2. Results

### 2.1. Identification of a MARCKS Antibody That Binds to the ED Peptide

First, a commercially available antibody against the MARCKS ED that binds to the ED peptide had to be identified so it could be used for competition ELISA. As a negative control, we used a control peptide with a scrambled amino acid sequence of the ED peptide, phosphate-buffered saline (PBS) as a vehicle control, and IgG isolated from non-immunized rabbits as an antibody control. At 2.5 µg/mL, the MARCKS antibody bound to the ED peptide in a concentration-dependent manner. MARCKS antibody binding reached saturation at 50 µM ED peptide (Figure S1). We lowered the concentration range in the assay to determine the binding of the MARCKS antibody and the shape of the curve more accurately in the 0–50 µM concentration range. Additionally, we reduced the ED peptide concentrations and used concentrations up to the 50 µM saturation point. The peptide reached saturation at around 10 µM (Figure S2). Based on these data, we decided to use the almost-saturating concentration of 5 µM ED peptide for the screen. As with the screen for L1 agonists [33], a concentration of 20 µM for the small organic compounds was used.

### 2.2. ED Peptide Mimetics Inhibited the Binding of ED Peptide to the MARCKS Antibody

**Figure 1:**
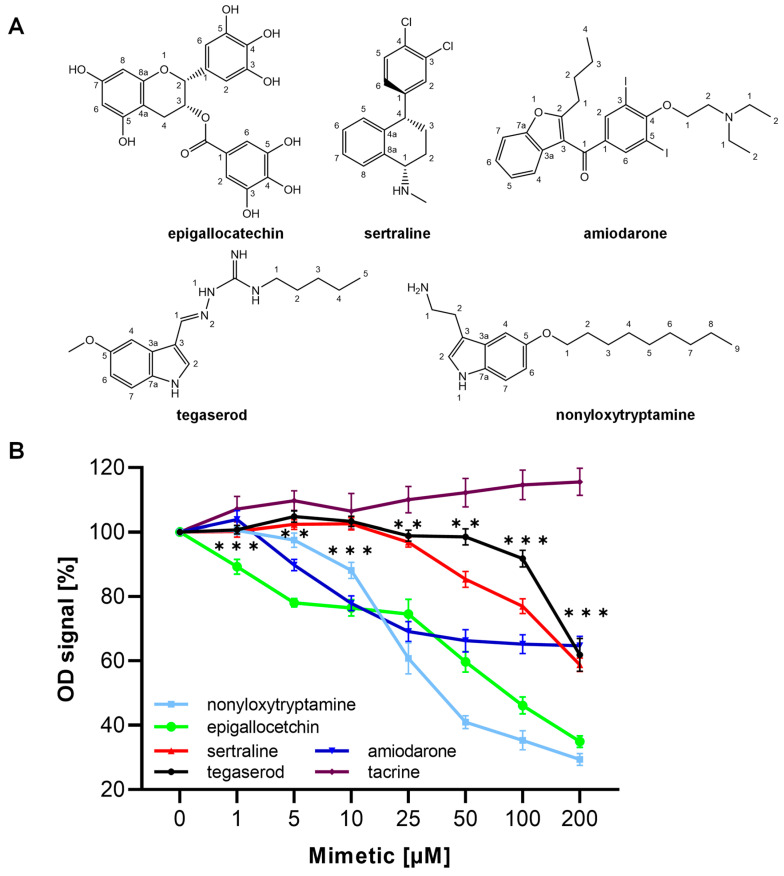
MARCKS ED peptide mimetics inhibited the binding of MARCKS antibody to the ED peptide. Chemical structures of identified mimetics (**A**). Binding of MARCKS antibody to the ED peptide in the presence of nonyloxytryptamine, epigallocatechin, sertraline, tegaserod, amiodarone, and tacrine (as a negative control). Data are represented as mean ± SEM (3 independent experiments carried out in triplicate). Asterisks show statistically significant differences between mimetics and negative control tacrine, ** *p* < 0.01, *** *p* < 0.001 (one-way ANOVA, F = 63.39, *p* < 0.0001) (**B**).

We identified five compounds (epigallocatechin, amiodarone, sertraline, tegaserod, and nonyloxytryptamine) that inhibited the binding of ED peptide to the antibody by at least 60% compared to the negative control. Small organic compound tacrine was chosen as the negative control. Being an L1 agonist mimetic [33], it did not bind to the MARCKS antibody. The five mimetics blocked ED peptide binding to the MARCKS antibody in a concentration-dependent manner. At 25 µM and higher molarities, all the mimetics significantly inhibited the ED peptide from binding to the antibody compared to tacrine (Figure 1), which indicates that the compounds are structurally similar to the region of the ED peptide to which the MARCKS antibody binds.

Next, we investigated whether PSA bound to the ED peptide in a concentration-dependent manner (Figure S3). We used a 10 µM concentration of PSA for the screen since PSA binding to the ED peptide is close to saturation at this concentration (Figure S3).

### 2.3. ED Peptide Mimetics Bound to PSA

**Figure 2:**
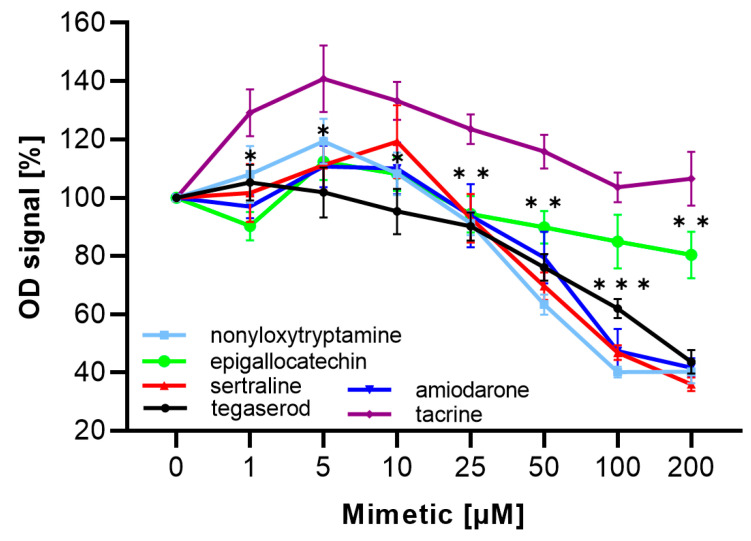
MARCKS ED peptide mimetics inhibited the binding of PSA to the ED peptide. The binding of PSA to the ED peptide was inhibited in a concentration-dependent manner by nonyloxytryptamine, epigallocatechin, sertraline, tegaserod, and amiodarone. Data are presented as mean ± SEM (3 independent experiments). Asterisks show statistically significant differences between epigallocatechin and the negative control tacrine; * *p* < 0.05, ** *p* < 0.01, *** *p* < 0.001 (one-way ANOVA, F = 12.512, *p* < 0.0001).

Furthermore, we used the competition ELISA to investigate if mimetics would interrupt the interaction between ED peptide and PSA and found that several mimetics inhibited the binding of PSA to ED peptide in a concentration-dependent manner (Figure 2). Only 10 µM sertraline and 100 µM epigallocatechin did not significantly compete with PSA for binding. These results show that the compounds mimicked the structure of the ED peptide.

In both ELISA assays, the tacrine exceeded 100%. In our previous study on mimetics of the HNK-1 carbohydrate [3], we used tacrine as a negative control compound. In the present test with the ED peptide, we had hoped it would also be negative in the competition ELISA, but it did not live up to our expectations. At higher concentrations, its effect became evident in comparison to the other compounds. We searched for other compounds that are similar in structure to the ED peptide mimetics but are negative in the competition ELISA but were not successful.

### 2.4. Effect of ED Peptide Mimetics on Neuronal Viability

**Figure 3:**
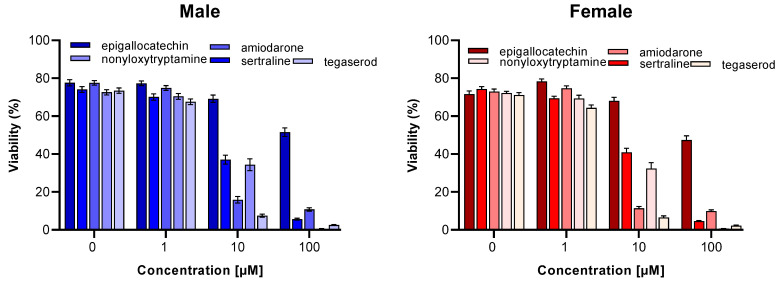
Effects of mimetics on neuronal viability. Cerebellar granule cells were incubated with ED peptide mimetics at different concentrations (1, 10, and 100 µM) in 0.1% DMSO (0 µM), which served as a vehicle control. Cell viability was measured by propidium iodide and calcein-AM staining. Data are presented as mean ± SEM of 3 independent experiments.

Mimetics were nontoxic for cerebellar granule cells from both sexes at 1 µM. A 10 µM concentration of epigallocatechin was also not toxic. We defined the toxic range to be any percent viability less than or equal to 50%. No significant sex-specific difference could be detected. Based on these toxicity experiments, we suggest using a maximum of 1 µM concentration in the in vitro functional assays (Figure 3). Neurite outgrowth assays were performed on male and female cerebellar granule cells treated with mimetics at concentrations up to 1 µM.

### 2.5. Effect of ED Peptide Mimetics on Neurite Outgrowth

**Figure 4:**
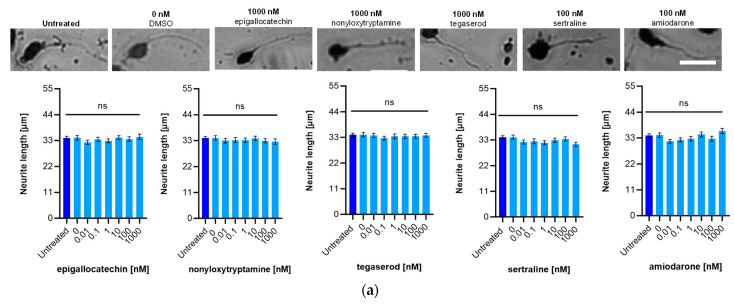

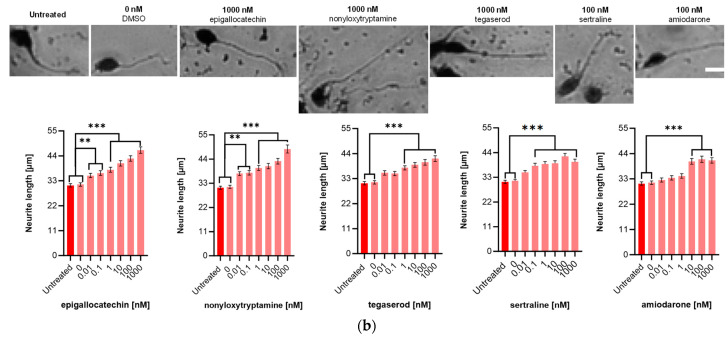
Effect of the ED peptide mimetics on neurite outgrowth. Cerebellar granule cells from male (**a**) and female (**b**) mice were seeded separately into 48-well plates. Cells were then incubated with 0.1% DMSO (0 nM) as vehicle control, and each mimetic was tested at final concentrations from 0.01 to 1000 nM in this vehicle. Data are presented as mean ± SEM (3 independent experiments). Shown are representative images of neurons from male (**a**) and female (**b**) mice at a concentration that induces maximum neurite outgrowth. Asterisks indicate differences between mimetics versus both untreated and DMSO (0 nM). ** *p* < 0.01, *** *p* < 0.001, ns = not significant. Scale bar = 20 µm.

Sex-specific neurite outgrowth was tested to analyze whether the mimetics would affect neurite outgrowth like the ED peptide [26]. We found no change (*p* > 0.05) in neurite outgrowth of male and female cerebellar granule cells between the untreated and dimethyl sulfoxide (DMSO) vehicle (0 nM) groups. There was also no change in neurite outgrowth with male (Figure 4a) cerebellar granule cells treated with different concentrations of mimetics. However, all mimetics stimulated neurite outgrowth in female cerebellar granule cells at 0.01–1000 nM, except for amiodarone, which promoted neurite outgrowth at only 10–1000 nM (Figure 4b). Nonyloxytryptamine was the most effective mimetic, promoting neurite outgrowth at 1000 nM (*p* < 0.001) with an average neurite length of 48.6 µm (SEM ± 1.85 µm) compared to the 0.1% DMSO vehicle (0 nM) control with an average neurite length of 31.4 µm (SEM ± 0.77 µm). Epigallocatechin, sertraline, and tegaserod also promoted neurite outgrowth with average neurite lengths of 46.5 µm (SEM ± 1.43), 42.2 µm (SEM ± 1.38), and 41.8 µm (SEM ± 1.27) at 1000, 100, and 1000 nM, respectively. Amiodarone was the least effective mimetic, yielding an average length of 41.5 µm (SEM ± 1.36) at 100 nM (Figure 4b).

Because we unsuccessfully used tacrine in the competition ELISA with the expectation that it would be a negative control, we did not include it in the in vitro functional tests since it promotes neurite outgrowth and neuronal survival [33].

## 3. Discussion

Previously, we found that the ED peptide enhances neurite outgrowth of cultured hippocampal neurons in an NCAM- and PSA-dependent manner [26]. We demonstrate and verify here that the ED peptide binds directly to PSA [26]. The present study identified small organic compounds that mimic the structure and function of the ED peptide by competition ELISA, thereby indicating the structural similarity to the ED peptide. Using competition ELISA in the past, we identified small organic compounds that structurally and functionally mimic the neural glycans LewisX [4], PSA [3,34,35,36], and HNK-1 [5]. By competition ELISA, we also previously found small compounds that function as agonists of the cell adhesion molecule L1 [33]. These compounds stimulate cellular functions that are essential for development, such as neurite outgrowth and neuronal survival. In support of the neuroprotective properties of these compounds, they are not neurotoxic, even at higher concentrations [3,4,5,37]. In contrast, the ED peptide triggers neurite outgrowth but is not neuroprotective when stressed with hydrogen peroxide [31]. Although our findings do not allow us to deduce that the ED peptide mimetics bind directly to MARCKS and its ED peptide, we propose that they associate: when extrapolating from previous parameters on ED- and MARCKS-related binding properties, direct binding of the mimetics is highly likely [26].

The ED peptide enhanced neurite outgrowth only in female mice cell culture, but it did not affect neurite growth in male mice [31]. Similarly, the present study reveals that ED peptide mimetics only stimulated neurite outgrowth of neurons from female mice. Interestingly, the ED peptide treatment also increased the phosphorylation levels of MARCKS in neurons from females only [31]. ED peptide mimetics have been used for the treatment of diseases and are also components of the human diet [38,39,40,41,42]. However, until now, they are not known to bind to PSA or are functional in a sex-specific manner. In the following paragraphs, we cite literature on ED peptide mimetics in vivo. The properties of these mimetics are compatible with our in vitro findings regarding their beneficial properties, such as neurite outgrowth and neuronal survival.

Epigallocatechin is the most abundant catechin in green tea and is of functional relevance since it reaches the brain parenchyma through the blood-brain barrier and induces neurogenesis [42]. It is neuroprotective in rodent models of Parkinson’s disease [43,44,45,46], Alzheimer’s disease [47,48], multiple sclerosis [49,50], and neurotrauma [51,52,53]. In addition, epigallocatechin displays anti-cancer properties [53]. This is interesting because the ED peptide also inhibits in vitro the cell migration of glioblastoma [54] and lung cancer [55]. Yet, these studies did not investigate sex differences. Our present study suggests that epigallocatechin may be more potent in females. In *Drosophila melanogaster*, epigallocatechin promotes sex-specific metabolic changes [56]. Females treated with epigallocatechin accumulate less fat, while males increase lean mass and locomotor activity [56].

Amiodarone is used to treat heart arrhythmias and is especially effective in patients with Wolff-Parkinson-White syndrome. It blocks ion channels in cardiac tissues, thus prolonging the cardiac action potential and refractory period [57]. Amiodarone is poorly bioavailable and undergoes extensive enterohepatic circulation. In addition, its terminal half-life is long and variable [40]. Regarding sex-specific differences in pharmacokinetics, amiodarone uptake by vascular tissue was higher in female than male mice but elicited an increase in vascular reactivity in only male mice [58]. In addition, the risk of bradyarrhythmia requiring pacemaker insertion associated with amiodarone use is higher in women than men [59].

Sertraline inhibits presynaptic reuptake of serotonin from the synaptic cleft. It is used to treat major depressive disorder, panic disorder, obsessive-compulsive disorder, and post-traumatic stress disorder [38]. Sertraline is slowly absorbed by the human body, well-tolerated in therapeutic dosages, and relatively safe in overdosage [38]. In transgenic mouse models for depressive-like disorders, females have an increased sensitivity to sertraline than males [60]. In addition, it is more effective in women compared to the antidepressant imipramine, whereas in men there is no difference between responses to sertraline and imipramine [61].

Tegaserod, also known as Zelnorm^®^, is a serotonin type 4 receptor agonist used to treat hypomotility disorders of the lower gastrointestinal tract associated with irritable bowel syndrome with constipation [39]. Tegaserod was approved by the FDA in 2002 but was subsequently removed from the market in 2007 due to FDA concerns about possible adverse cardiovascular effects. However, a matched case-control study of tegaserod-treated with untreated patients found no association between tegaserod and adverse cardiovascular outcomes [62]. Thus, it was resubmitted to the FDA in 2018 for use in a low-risk population [63]. Although only one study reported that tegaserod was more efficient in men to accelerate gastric emptying, the authors suggest that tegaserod is a potent prokinetic agent throughout the gastrointestinal system in both sexes [64]. Of note in this context is that we have identified tegaserod as a mimetic for the PSA as well [34].

Nonyloxytryptamine is a selective agonist of the 5-HT1B receptor [41], and we identified it previously as a mimetic for the neural glycans PSA [65] and HNK-1 [5]. It stimulates beneficial functions such as neurogenesis in hippocampal neurons and motoneurons and the migration of Schwann cells, as well as improves functional recovery after spinal cord injury [36]. Yet, potential sex differences in functional recovery were not analyzed in this study. However, we found that estrogen receptors α and β are essential for ED-peptide-enhanced neurite outgrowth in female neurons. In addition, the ED peptide enhanced neurite outgrowth in male neurons when the androgen receptor was inhibited. The ED peptide also enhanced the level of MARCKS phosphorylation in female neurons. However, this was not observed in male neurons after the androgen receptor was inhibited. This indicates that a different molecular mechanism underlies ED-peptide-enhanced neurite outgrowth in male versus female neurons [31]. Their sex differences in protein kinase C activity and isozyme expression may indicate that sex differences in MARCKS phosphorylation could contribute to the dependence on differences in PKC activity between the sexes [66,67].

Different small molecule structures bind to the ED peptide antibody and PSA, as similarly observed with small molecules that we identified to mimic the glycans PSA, LewisX, and HNK-1 and the cell adhesion molecule L1. These mimetics specifically triggered the signaling pathways triggered by PSA, LewisX, HNK-1, or L1, although these mimetics were structurally different [1,2,3,37]. We infer that the smaller sizes of the mimetics allow for a more flexible binding into the pockets of the specific receptors. For example, computational modeling of PSA and the PSA mimetic tegaserod as published for the structure of the monoclonal PSA antibody 735 revealed that an eight-residue PSA fragment forms a half helical turn in the binding pocket of the antibody. PSA thus binds to a broad groove in the antibody binding pocket, whereas tegaserod binds within the pocket in only one region of the putative PSA binding site [34]. Thus, different small molecules may bind either at a different position in the larger binding pocket of the antibody or with different affinities to the antibody, so that molecules function differently in females and males as seen for the ED peptide mimetics of MARCKS. We also show that these mimetics target PSA and have shown that different small molecule structures can bind to the same receptor. It is not clear if the binding of PSA to the different mimetics may play a role in the described sex-specific effects of the ED peptide mimetics. It is noteworthy in this context that the androgen receptor blocks the ED peptide’s influence on neurite outgrowth. Hence, we would like to suggest a similar mechanism for the ED peptide mimetics. Considering that PSA is an important player in cell functions outside the nervous system [67], it would be beneficial to optimize treatments with the mimetics in animal disease models in vivo with a view to therapy in human females. Whether small compound treatments with mimetics for men can be found remains to be seen.

## 4. Materials and Methods

### 4.1. Animals

To obtain six- to eight-day-old (P6-P8) mice for experiments, we used ten-week-old CB6F1/J mice (Strain #:100007, Jackson Laboratory, Bar Harbor, ME, USA) for breeding, and we maintained them (12:12 h; light: dark cycle) in the animal facility of the Division of Life Sciences at the Nelson Biology Laboratories of Rutgers University. They were provided with ad libitum access to food and water. We separately used male and female offspring for the preparation of primary cerebellar granule cell culture. The experiments followed ARRIVE guidelines.

### 4.2. Antibodies and Small Compounds

ED peptide antibody (cat# SAB4300511, Lot 951421257) was from Sigma-Aldrich (St. Louis, MO, USA). ED-MARCKS N-terminus biotinylated peptide (ED peptide; Biotin-KKKKKRFSFKKSFKLSGFSFKKNKK; molecular weight: 3307 g/mol; cat# U5987EB150-1/PE0714) and ctrl-MARCKS N-terminus biotinylated peptide (control peptide) were from GenScript (Piscataway, NJ, USA). (Z)-but-2-enedioic acid;1-[(5-methoxy-1H-indol-3-yl)methylideneamino]-2-pentylguanidine (tegaserod; CAS 189188-57-6; cat# SML1504-10MG), 2-(5-nonoxy-1H-indol-3-yl)ethanamine;oxalic acid (nonyloxytryptamine; CAS 157798-13-5; cat# SML1255-5MG), [(2R,3R)-5,7-dihydroxy-2-(3,4,5-trihydroxyphenyl)-3,4-dihydro-2H-chromen-3-yl] 3,4,5-trihydroxybenzoate (epigallocatechin; CAS 989-51-5; cat# E4143-50MG), (1S,4S)-4-(3,4-dichlorophenyl)-N-methyl-1,2,3,4-tetrahydronaphthalen-1-amine;hydrochloride (sertraline; CAS 79559-97-0; cat# S6319-10MG), (2-butyl-1-benzofuran-3-yl)-[4-[2-(diethylamino)ethoxy]-3,5-diiodophenyl]methanone;hydrochloride (amiodarone; CAS 19774-82-4, cat# A8423-1G), colominic acid sodium salt from *Escherichia coli* (Poly [2,8-(N-acetylneuraminic acid sodium salt)]; PSA; CAS 70431-34-4; cat# C5762-100MG), and poly-lysine-L (PLL; CAS 25988-63-0 Corning; cat# 3548) were from Sigma-Aldrich. These and the following small organic compounds were dissolved in dimethyl sulfoxide (DMSO; CAS 67-68-5; cat# 20-139, Sigma-Aldrich). Ortho-phenylenediamine dihydrochloride (OPD, CAS 615-28-1), acetyloxymethyl 2-[[2-(acetyloxymethoxy)-2-oxoethyl]-[[3′,6′-diacetyloxy-7′-[[bis[2-(acetyloxymethoxy)-2-oxoethyl]amino]methyl]-3-oxospiro[2-benzofuran-1,9′-xanthene]-2′-yl]methyl]amino]acetate (calcein-AM; CAS 148504-34-1; cat# C1430; LOT 2103356), and mouse monoclonal antibody against the neural cell adhesion molecule NCAM-1/CD56 antibody (anti-NCAM 735; cat# NBP2-52710; LOT T1521A03) were from Thermo Fisher Scientific (Waltham, MA, USA). Peroxidase-conjugated streptavidin (cat# 016-030-084) and AffiniPure Donkey Anti-Mouse IgG (H + L) coupled to horseradish peroxidase (HRP) (cat# 715-035-151) were from Jackson ImmunoResearch (West Grove, PA, USA). Pierce™ Streptavidin-Coated High-Capacity Plates (cat# 15500) and Hank’s balanced salt solution (HBSS) (cat# 14175-095) were purchased from Thermo Fisher Scientific. X-1 media was made using Neurobasal A medium (cat# 10888022) and supplemented with 1% penicillin–streptomycin (cat# 15140148), 1% sodium pyruvate (cat#11360070), 1% B-27 supplement (cat# A3582801), and 1% GlutaMAX (cat# 35050061), all purchased from Thermo Fisher Scientific. ViaStain AO/PI (acridine orange/propidium iodide) staining solution for live/dead mammalian nucleated cells (cat# CS2-0106-5mL) was from Nexcelom Bioscience (Perkin Elmer, Lawrence, MA, USA).

### 4.3. Enzyme-Linked Immunosorbent Assay (ELISA) to Screen for ED Peptide Mimetics

To identify mimetics of ED peptide, we screened small organic compounds as described [68] using the National Institute of Health Clinical Collection Libraries 1 and 2 (Evotec, Marburg, Germany) and the Natural Compound Library (Selleckchem, Houston, TX, USA) via competition ELISA. In brief, 384-well ELISA plates were coated with 1 µg/mL of antibody against ED. For control, two wells per plate were treated with phosphate-buffered saline, pH 7.3 (PBS). The wells were incubated overnight at 4 °C, then treated with blocking solution (5% bovine serum albumin (BSA) + 0.1% Triton X-100 in PBS) for 1 h at room temperature on a shaker (200 rpm; Orbi-Shaker™ Jr., Benchmark Scientific, Sayreville, NJ, USA). All subsequent incubations on the shaker were at the same rpm. Thereafter, 25 µL of 40 µM of each compound from the compound libraries was added and incubated at room temperature for 30 min. Each treatment was performed in duplicate. Subsequently, 25 µL of 10 µM N-biotinylated ED peptide was mixed with the compounds in each well (for final compound and peptide concentrations of 20 µM and 5 µM, respectively) and incubated for 1 h at room temperature. Wells were then washed three times with PBS and treated with streptavidin coupled to HRP at 1:5000 in 5% BSA blocking solution. Wells were then incubated for 30 min at room temperature. Afterward, wells were washed three times with PBS and then treated with HRP substrate (0.1% o-phenylenediamine dihydrochloride (OPD) + 0.1% hydrogen peroxide (H_2_O_2_) in OPD buffer). After 20 min, we stopped the reaction with 2.5 M sulfuric acid and read the absorbance at 450 and, for the wavelength correction, at 570 nm with an ELISA plate reader (EL800; BioTek Instruments, Winooski, VT, USA). Compounds that were active by competition in the ELISA were tested for their ability to compete with the ED peptide for the binding to ED antibody in a concentration-dependent manner. To this aim, we repeated the competition ELISA with the following modifications: After blocking, we added different concentrations (0, 1, 5, 10, 25, 50, 100, and 200 µM) of the identified compounds to the wells. Each experiment was performed in triplicate and was repeated three times independently.

To determine if the ED peptide mimetics inhibit the binding of MARCKS to PSA, we first incubated different concentrations (0, 1, 5, 10, 25, 50, 100, and 200 µM) of each mimetic with 20 µg/mL of PSA at 4 °C overnight. The mimetics and PSA were added in equal volumes. Then, we coated 5 µM N-biotinylated ED peptide in a 96-well, streptavidin-coated plate and incubated it for 1 h at room temperature. Subsequently, we removed the ED peptide from the wells and added 50 µL of the mixed solution containing PSA and small organic compounds to incubate at room temperature for 1 h. Afterwards, we removed the solution, washed the wells 3 times with PBS, and added 50 µL of 1:200 anti-PSA (monoclonal antibody 735) diluted in PBS to incubate for 1 h at room temperature. Subsequently, we removed the primary antibody and washed the wells 3 times with PBS before adding 1:1000 of anti-mouse IgG coupled to HRP in 0.3% Triton X-100 and 5% BSA plus PBS. The wells were then incubated at room temperature for 30 min before signal detection with the ELISA plate reader as described above.

### 4.4. Cerebellar Granule Cell Culture

The sex of young mice was determined using anogenital distance and the presence of inguinal mammary teats [69]. Male and female cells were prepared separately. Cerebellar granule cell cultures were prepared as described [70]. Briefly, cerebella were incubated in 0.04% of trypsin, 4% of DNase I, and 0.8 mM of MgCl_2_ in HBSS for 15 min at 37 °C and mechanically dissociated. Dissociated cells were mixed with ViaStain™ AOPI Staining Solution, prepared, and counted according to Cellometer Auto 2000 Cell Viability Counter (Nexcelom) manufacture information. Subsequently, dissociated cells were seeded on 48-well plates coated with 0.01% PLL.

### 4.5. Toxicity Assay

Compounds were tested for toxicity as described [31]. Briefly, after counting the dissociated cells, they were diluted to 125,000 cells/well with pre-warmed X-1 medium on a PLL-coated 48-well plate and maintained at 37 °C for 24 h. Cells were then incubated for 24 h with 1, 10, and 100 µM of the mimetics in 0.1% DMSO, which was used as vehicle control. For live imaging, propidium iodide and calcein-AM solution (each 4 ng/mL) were first added to each well, and the plate was then incubated (37 °C) for 20–30 min. Images of live and apoptotic cells were captured with a Zeiss Axiovert 200 M inverted light transmission microscope using a 20× objective (Carl Zeiss, Oberkochen Germany). The AxioVision 4.6 software was used to control contrast and adjust the clarity of the images (Carl Zeiss, NY, USA). Four images were taken per well (12 images/condition). The experiment was performed three times independently and in triplicates for each compound (*n* = 36). The percentage of viable cells was calculated by the ratio between calcein-positive cells and the total cell number.

### 4.6. Neurite Outgrowth

Neurite outgrowth was measured as described [65]. In brief, the cerebellar granule cells were dissociated and after counting, cells were diluted to 100,000 cells/mL with pre-warmed X-1 medium and seeded onto 0.01% PLL-coated 48-well plates. Immediately after being seeded, cells were stimulated with 0, 0.1, 1, 10, 100, and 1000 nM of each mimetic in 0.1% DMSO. Plates were then incubated for 24 h at 37 °C. To fix cells, wells were treated with 2.5% glutaraldehyde and incubated for 30 min at room temperature. Subsequently, the wells were washed with distilled water three times. A total of 100 µL of staining solution (1% toluidine blue O and 1% methylene blue in 1% borax) was added to the wells and they were incubated at room temperature for 30 min. The wells were washed three times with distilled water. The plates were then dried, and 6–8 images were taken per well using a Zeiss Axiovert 200 M and the AxioVision 4.6 software. At least 100 cells with neurites of at least twice the length of the cell body were traced and measured using ImageJ (Version 1.50b; National Institutes of Health, Bethesda, MD, USA). Compounds were tested in three independent experiments (*n* = 300).

### 4.7. Statistical Analysis

Data are presented as mean ± standard error of the mean (SEM) from 3 experiments unless indicated otherwise in the figure legends. Statistical comparisons between groups were performed by one-way analysis of variance (ANOVA) followed by post hoc Fisher’s least significant difference test using GraphPad Prism 9.5.0 (Boston, MA, USA). Chemical drawings were prepared with ChemDraw Professional 16.0 Suite (Perkin Elmer, Waltham, MA, USA).

## Data Availability

The datasets used and/or analyzed during the current study are available from the corresponding authors upon reasonable request.

## Supplementary Materials

The supporting information can be downloaded at https://www.mdpi.com/article/10.3390/ijms241814271/s1.
