# Supplementary material for: Small Organic Compounds Mimicking the Effector Domain of Myristoylated Alanine-Rich C-Kinase Substrate Stimulate Female-Specific Neurite Outgrowth
Source: Int J Mol Sci. 2023 Sep 19;24(18):14271. doi: 10.3390/ijms241814271 (PMC10532424; doi:10.3390/ijms241814271)
Supplement: Supplementary file 1 [file ijms-24-14271-s001.zip › ijms-2575346-supplementary.pdf]

Supplementary Materials

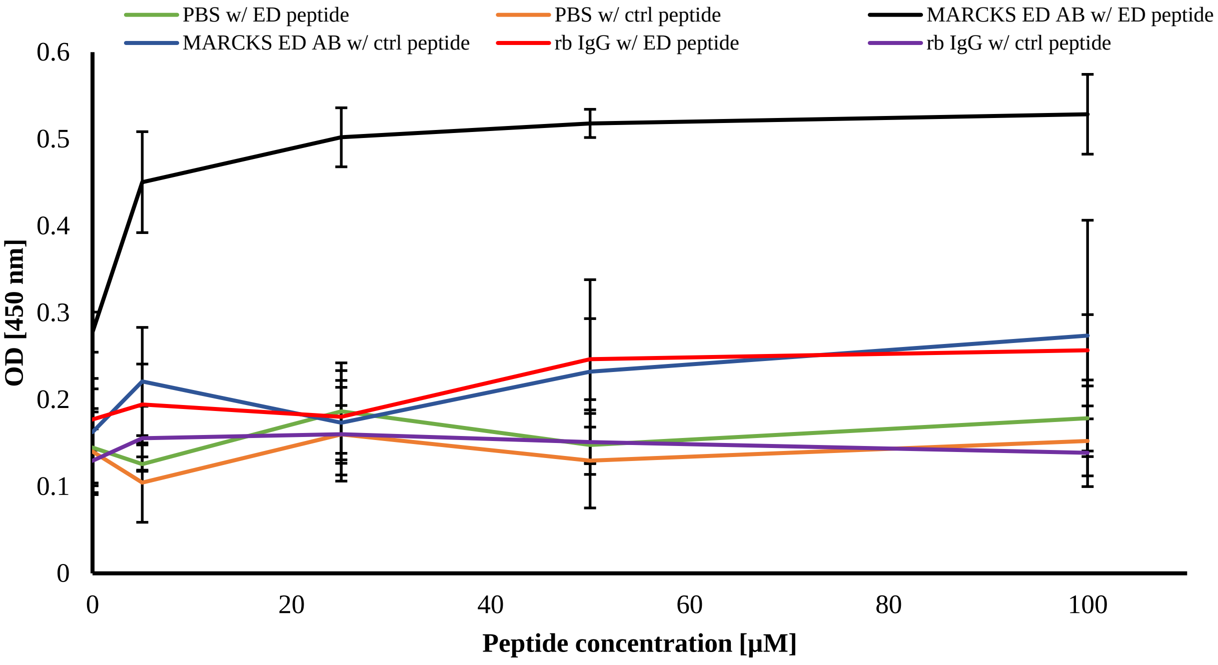

Figure S1. Identifying MARCKS antibody that binds to the MARCKS ED peptide. Non-treated ELISA plates were coated with either rabbit antibody that binds to the effector domain of MARCKS (MARCKS antibody), control antibody from rabbit serum (IgG rabbit control), or only PBS. Wells were then treated with different concentrations (0, 5, 25, 50, 100, and 200 µM) of the N terminus biotinylated MARCKS ED peptide or the scrambled control peptide. Peptides were then detected with streptavidin coupled to horseradish peroxidase (HRP) and optical density (OD) was measured at a wavelength of 450 nm. This experiment was performed once. Error bars represent the standard error of the mean.

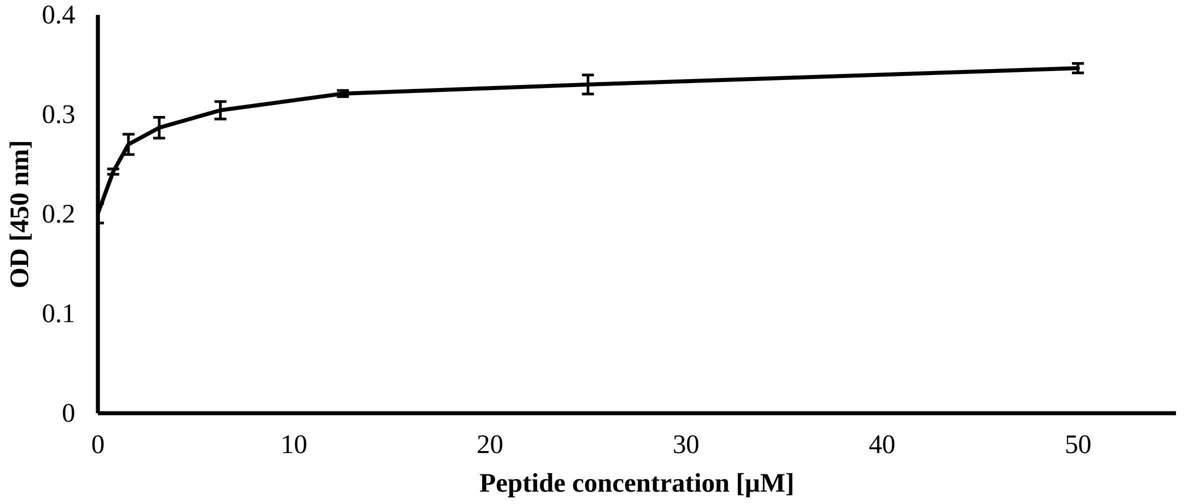

Figure S2. Determining if MARCKS antibody binds to lower concentrations of MARKCS ED peptide in a concentration-dependent manner. Non-treated ELISA plate was coated with 2.5 µg/ml of rabbit antibody that binds to the effector domain of MARCKS (MARCKS antibody). Wells were then treated with different concentrations (0, 0.78, 1.56, 3.13, 6.25, 12.5, 25, and 50 µM) of the N terminus biotinylated MARCKS ED peptide. This peptide was then detected with streptavidin coupled to horseradish peroxidase (HRP) and optical density (OD) was measured at 450 nm. This experiment was performed in two independent trials and data from these trials were merged into the graph above.

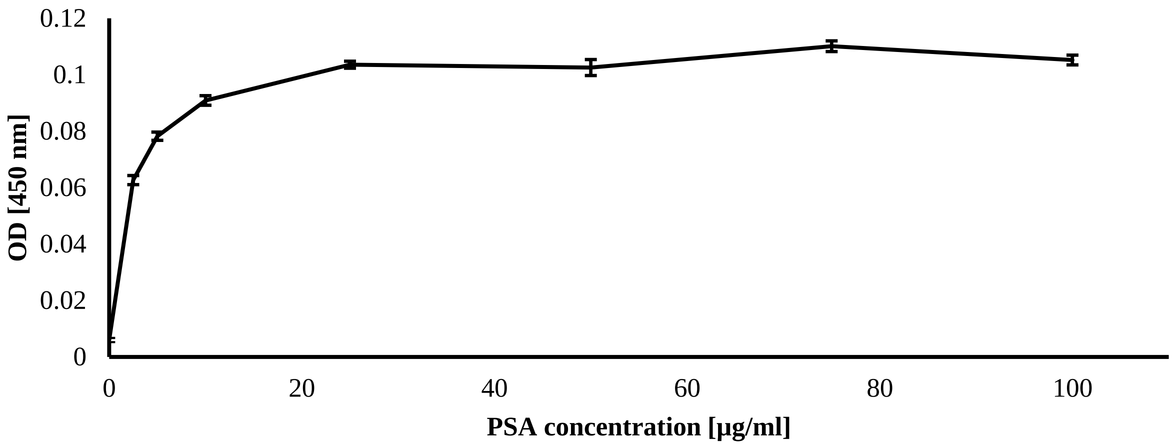

Figure S3. PSA binds to the MARCKS ED peptide in a concentration-dependent manner. A 96-well ELISA plate pre-coated with streptavidin was coated with 5 µM of biotinylated MARCKS ED peptide. Wells were then treated with different concentrations (0, 2.5, 5, 10, 25, 50, 75, and 100 µg/mL of PSA. PSA was then detected with Anti-NCAM PSA mouse antibody (735) and secondary mouse antibody coupled to horseradish peroxidase (HRP) optical density was calculated by subtracting values measured from a wavelength of 570 from values measured from a wavelength of 450. Data are presented as mean ± SEM (n = 9, 3 independent experiments).
